# Is the Little Toe Disappearing?

**Published:** 1900-11

**Authors:** 


### IS THE LITTLE TOE DISAPPEARING?

When primitive man swung from limb to limb to escape the assault of more formidable carnivora and slept on his back in a lofty nest of sticks like his quadrumanous prototype the orang-outang, or led the hard life of the troglodyte, he was probably covered with hair. With the first weapons came the means of killing game, and there was then an easy transference of the skin of the animal to the back of man. Thus came about the notion of artificial protection for the body, and clothes became a point of differentiation of man from other animals. For a more extended notice of the origin of clothes the reader is referred to the lucubrations of that astute philosopher Herr Teufelsdröck. As to how long the possession of hair survived its functionless desuetude, history is silent. But it went, and except for a few sports of nature here and there man is practically a glabrous animal, and as he ascends in the scale of development, he becomes more and more freed from this pithycoid relic. At least this is the argument of the prematurely bald, and, whether fallacious or not, perhaps the thought may be of some comfort to them. The foreskin is said to be an example of evolutionary remains, and its absence is taken as an instance of the disappearance of an organ which has lost its function. A similar theory has been advanced for that malignant cecal appenage, the vermiform appendix.

The application of these facts of more or less general acceptance lies in a portion of the body which must have undergone change commensurate with the abridgment of function—the feet, or more properly the toes. The feet of the horse, the cow, and other even-and odd-toed animals have undergone great alteration to establish the balance between organ and function which must needs be adjusted in working out the great principle of design.

To apply the same theory of pedal evolution to man is a short and perfectly legitimate step forward.

Man must have lost very early the prehensile power of the toes. In fact Lombroso regards the persistence of this quality as a reachback to the primitive type.

When with improved instruments of offense and defense man was able to prevail in the struggle for existence against savage and predatory animals, the necessity for his taking "to the tall timber"

when pursued by them was rare, and hence the grasping power of the toes disappeared along with his hair and his climbing muscles. Then too he began to wear foot gear, the forerunner of the modern boot. It would be an interesting study to follow the development of foot covering from the sandal to the present form which conventionality, backed by very unanatomical shoemakers, assumes to dictate. But it is not germane to our purpose further than to note that the boot has become tighter as civilization has advanced. The effect of this has been a widening separation of the analogy between the hands and the feet, once very close. The movements of the toes are curtailed by resting on a more or less inelastic sole and are further hampered by the lateral constriction of the shoe. The law of parsimony in nature, ably assisted by modern Crispins, is tending to eliminate the least effective and most exposed digit. Gnarled with corns, incurved, warped, with rudimentary nail or none at all, can it be doubted that man is losing his little toe? Who in these Procrustean days of shoes can "show up" an extremity that could call forth that burst of admiration, "Thy feet are beautiful upon the hills, O Benjamin! Thy feet are beautiful." Unless the signs of the times be misread, or there be a saving reversion to the original type, the man of the future will be a three- or four-toed animal, and perhaps that singular affection ainhum is to be interpreted as a *tour de force* of nature to abbreviate the course of a slowly acting law. The little toe is degenerate and must die.

